# Rapid signaling reactivation after targeted BRAF inhibition predicts the proliferation of individual melanoma cells from an isogenic population

**DOI:** 10.1038/s41598-021-94941-8

**Published:** 2021-07-29

**Authors:** Payam Khoshkenar, Emily Lowry, Amir Mitchell

**Affiliations:** 1 Program in Systems Biology, University of Massachusetts Medical School, Worcester, USA; 2 Program in Molecular Medicine, University of Massachusetts Medical School, Worcester, USA; 3 Department of Molecular, Cell and Cancer Biology, University of Massachusetts Medical School, Worcester, USA

**Keywords:** Wide-field fluorescence microscopy, Oncogene proteins, Tumour heterogeneity, Growth factor signalling, Cellular imaging, Cellular noise, Single-cell imaging, Time series, Cellular signalling networks

## Abstract

Cancer cells within tumors display a high degree of phenotypic variability. This variability is thought to allow some of the cells to survive and persist after seemingly effective drug treatments. Studies on vemurafenib, a signaling inhibitor that targets an oncogenic BRAF mutation common in melanoma, suggested that cell-to-cell variation in drug resistance, measured by long-term proliferation, originates from epigenetic differences in gene expression that pre-exist treatment. However, it is still unknown whether reactivation of signaling downstream to the inhibited BRAF, thought to be a key step for resistance, is heterogeneous across cells. While previous studies established that signaling reactivation takes place many hours to days after treatment, they monitored reactivation with bulk-population assays unsuitable for detecting cell-to-cell heterogeneity. We hypothesized that signaling reactivation is heterogeneous and is almost instantaneous for a small subpopulation of resistant cells. We tested this hypothesis by monitoring signaling dynamics at a single-cell resolution and observed that despite highly uniform initial inhibition, roughly 15% of cells reactivated signaling within an hour of treatment. Moreover, by tracking cell lineages over multiple days, we established that these cells indeed proliferated more than neighboring cells, thus establishing that rapid signaling reactivation predicts long-term vemurafenib resistance.

## Introduction

Numerous studies over the past decade revealed extensive diversity between cancer cells within tumors. These cell-to-cell differences, often referred to as heterogeneity, can involve multiple cellular pathways and underly significant phenotypic variation. In some cases, cell-to-cell heterogeneity can engage key oncogenic pathways underlying disease progression or influencing treatment resistance and can therefore pose significant challenges for personalized cancer treatment^1^. Cell-to-cell variation is typically thought to originate from genomic instability leading to genetic differences between individual cancer cells within a tumor. However, multiple recent studies have also revealed that non-genetic mechanisms can play a significant role in promoting heterogeneity with potential important implications for correct diagnostics, disease progression and treatment options^2–6^. In these cases, isogenic cancer cells exist in alternative epigenetic states that arise from transcriptional and post-transcriptional differences between them^7,8^. Since the underlying mechanisms driving this type of heterogeneity can be independent of genetic differences (mutations) between cells, an individual cell can readily transition between two or more phenotypic states^5,6^.

The research of epigenetic-driven heterogeneity has greatly benefitted from in-vitro experiments in cell-line cultures. Such experiments, especially with clonal cell-lines that were derived from a single ancestor cell, allow controlling for multiple parameters that are highly variable in the extracellular tumor microenvironment and can also contribute to heterogeneity. Such parameters include uneven nutrient availability, oxygen gradients and spatial pH differences across the tumor environment^9–11^. Hence, in-vitro experiments allow for elucidation and focus on the inherent phenotypic diversity that is still retained even in a highly regulated and uniform in-vitro environment^4–6^. Melanoma cell-lines harboring the oncogenic BRAF^V600E^ mutation have become a primary model system for studying adaptive drug resistance and epigenetic-driven heterogeneity^5,12–14^. Specifically, the BRAF^V600E^ mutation, which is the most frequent BRAF mutation in melanoma, stimulates the constitutive activation of the downstream extracellular signal-regulated kinase (ERK). Constitutive activation of this pro-proliferative kinase in tumor cells, even in the absence of any extracellular stimuli, allows cells to become self-sufficient in growth signals^15^. Vemurafenib, the first drug approved for BRAF-mutant cancer, is a selective inhibitor of oncogenic BRAF that offers a significant clinical benefit for patients with metastatic melanoma^16^. Vemurafenib acts by selectively binding to the mutated BRAF protein which in turn inhibits the downstream mitogen-activated protein kinase network, including ERK.

**Figure 1:**
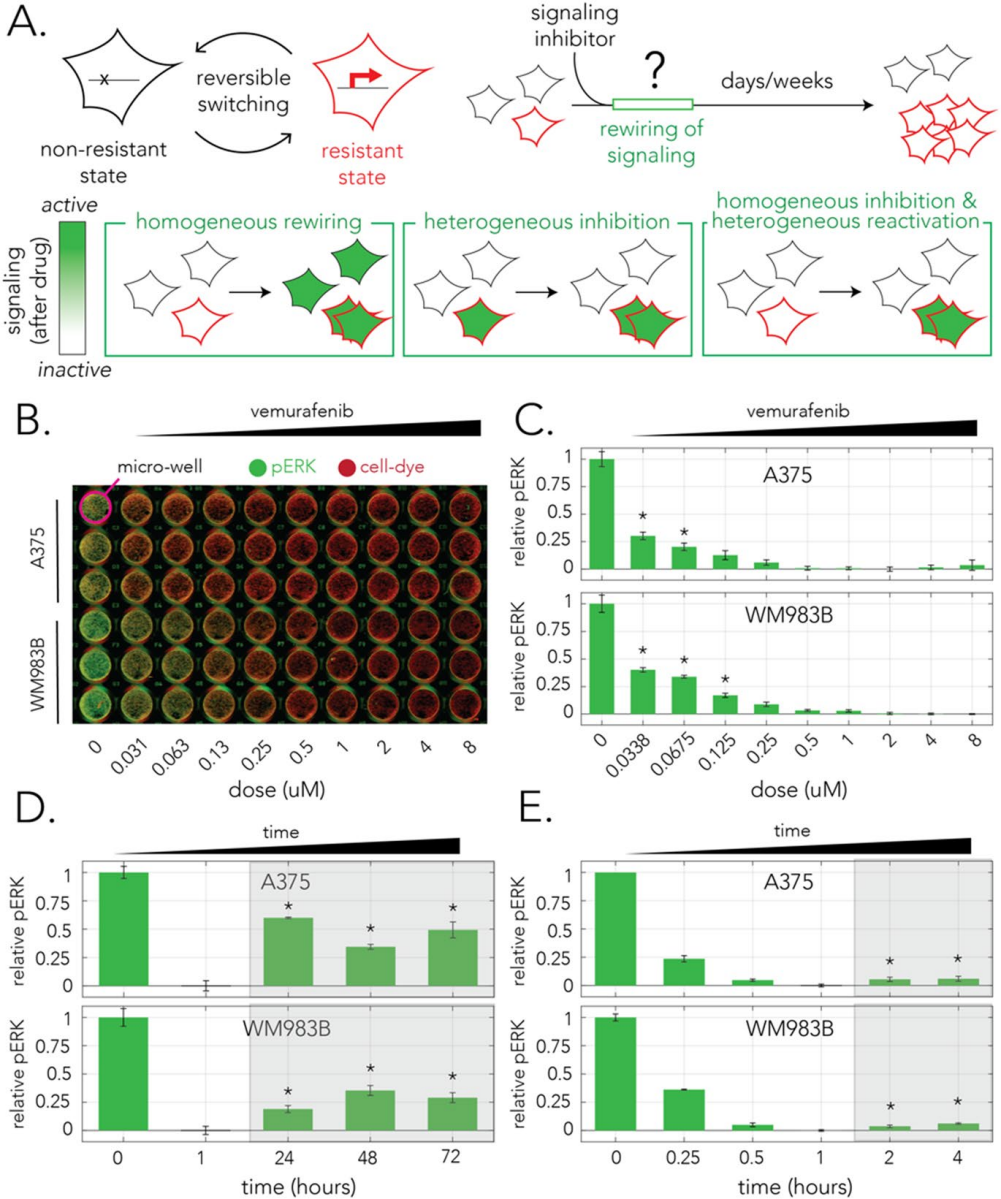
Reactivation of ERK signaling after vemurafenib treatment in bulk populations. (**A**) The epigenetic-based model for drug resistance and alternative models for signaling rewiring. High expression of a set of genes is thought to underlie a reversible drug-resistant state (upper-left panel). A small subpopulation of drug-resistant cells proliferates more than kin-cells after drug administration, however, the dynamics of signaling rewiring immediately after drug treatment in individual cells remains unknown (upper-right panel). The lower panels show three alternative models for signaling rewiring consistent with measurements from bulk populations. (**B**) A representative image of the immunofluorescence signal in the in-cell-western (ICW) method used for monitoring signaling activity 1 h after treatment. Cells growing in a micro-well plate were fixed and stained with an antibody specific for phosphorylated ERK (green). The wells were co-stained with a cell-dye (red) to monitor population size. The ratio between the two signals (green/red) marks the ERK activity normalized to cell number. (**C**) Vemurafenib impact on ERK signaling at different concentrations. The bars mark the relative pERK levels 1 h after treatment. (**D**) Vemurafenib impact on ERK signaling after multiple days of treatment. In both cell-lines, signaling recovers and rebounds to intermediate levels already within the first 24 h (reactivation period is marked by shaded area). (**E**) Vemurafenib impact on ERK signaling after multiple hours of treatment. In both cell-lines, signaling inhibition is maximized 1 h after treatment and then marginally recovers within 2 h (shaded area). In all graphs (**C–E**), green bars mark the relative pERK levels after treatment averaged over 3 biological replicates and thin black bars mark standard deviation. The relative ERK activity is calculated by comparing its normalized level to the minimum and maximum levels observed. Stars mark pERK levels that are statistically higher than the maximum inhibition observed (one-tailed t-test with a Bonferroni corrected *p*-value for multiple statistical tests).

While vemurafenib emerges as a highly effective anti-cancer treatment reaching 80% tumor response rate among patients, its potency is typically only transient, and after initial response, drug resistance arises in most patients^17^. A recent pioneering study that investigated vemurafenib resistance in-vitro in isogenic populations of melanoma cells revealed that drug resistance can arise from a reversible epigenetic state that characterizes a rare subpopulation of cells^5^ (Fig. 1A). The study leveraged on cell cultures that were plated sparsely for monitoring the long-term fate of individual cells and revealed that resistance is extremely heterogeneous and is correlated with high expression of multiple resistance marker genes, many of which belong to the gene network responding to extra-cellular growth factors (e.g., the growth factor receptors NGFR and EGFR).

The recent observations of drug resistance in a rare cell population through semi-coordinated high expression of specific genes, such as growth factor receptors, agree with previous seminal work that revealed that rewiring of signaling downstream to BRAF is a key step underlying adaptive resistance^13^. This study tested vemurafenib efficacy in diverse BRAF^V600E^ mutated cell-lines and showed that although vemurafenib was initially efficient in inhibiting downstream signaling, the shutdown was only transient. Within 24–48 h of drug administration, ERK phosphorylation levels rebound until they stabilized on a new intermediate steady-state level. ERK reactivation was suggested to originate from rewiring of the upstream signaling network that relieves an ERK-dependent negative feedback that suppresses ligand-dependent signaling in untreated cells. The relief of ERK-dependent negative feedback reactivates signal transduction through the generation of vemurafenib-resistant RAF dimers and culminates in renewed cell proliferation. In agreement with this mechanism of rewiring, co-administration of a MEK inhibitor with the targeted BRAF inhibitor mitigated signaling reactivation^13^. Additional observations showing that stromal cell secretion of hepatocyte growth factor can elicit resistance against RAF inhibitors further support the model of resistance through ligand-dependent signaling upon treatment^18^.

Given the widely accepted model of signaling reactivation and the discovery of a rare cell population that is drug resistant, an important open question that arises is therefore how signaling reactivation transpires at the single-cell resolution. However, since most studies that investigated signaling rewiring relied on bulk assays, methods that pool large cell populations together for measurements, the dynamics of signaling rewiring at the single-cell resolution remain elusive. The diagram in Fig. 1A shows three alternative models of single-cell behaviors that are consistent with existing observations of signaling reactivation in bulk population assays. Under the *homogenous model* (Fig. 1A, lower-left panel) signaling rewiring is homogenous across all cells irrespective of their drug resistance state (only resistant cells later continue to proliferate). Under the alternative models, cell behavior is heterogeneous. In one heterogeneous model (Fig. 1A, lower-middle panel), cell-to-cell variation already emerges at the initial signaling inhibition phase. In this model signaling in resistant cells remains unaffected by the drug and a profile of signaling reactivation seemingly arises since un-inhibited cells continue to proliferate and gradually increase their proportion in the population. In a second heterogeneous model (Fig. 1A, lower-right panel), all cells are initially inhibited by the drug, but resistant cells rapidly reactivate signaling.

In this study we examined how changes in ERK signaling transpire upon treatment at the single-cell resolution and tested how signaling dynamics in individual cells correlate with their ultimate proliferation capacity (drug resistance). Given observations that resistant cells highly express multiple growth factor receptors prior to drug administration^5^, we hypothesized that rewiring of signaling will take place almost instantaneously for a small sub-population of cells (following the homogenous inhibition, heterogeneous reactivation model in Fig. 1A). We reasoned that this prediction can be tested by monitoring signaling dynamics within the first few hours of treatment using a live-cell signaling reporter. We further predicted that single-cell measurements will allow us to observe signaling reactivation much earlier than was previously reported in the literature since previous studies relied on pooled population measurements that “average out” heterogeneous cellular responses. Lastly, we tested if signaling dynamics immediately after treatment are good predictors of ultimate cell fate (drug resistance). We tested our predictions using a combination of bulk-population measurements and high-throughput time-lapse microscopy experiments. These approaches allowed us to monitor signaling dynamics in thousands of individual cells and rigorously quantify cell–cell heterogeneity. In agreement with our prediction, we observed that despite uniform initial inhibition, early reactivation of ERK signaling is observed within an hour for a small subpopulation of cells and that these cells indeed proliferate more than non-recovering neighboring cells after multiple days of drug treatment. We concluded that the identification of a rapidly recovering subpopulation supports the hypothesis that a small fraction of melanoma cells pre-exists in a drug-resistant state and reconciles the widely accepted model for drug recovery through rewiring of signaling with recent observations made in single cells.

## Results

### Bulk measurement of signaling inhibition and reactivation

The effect of BRAF inhibition on MAPK signaling is typically monitored by testing the phosphorylation state of the downstream kinase ERK with semi-quantitative biochemical methods, such as Western blots, that measure the phosphorylation state averaged over large cell populations (e.g.^13,19^). In order to similarly quantify ERK phosphorylation levels (pERK) in bulk populations we used the in-cell-Western (ICW) method previously used by us and others to monitor ERK signaling^20^. Figure 1B shows a representative image of an ICW assay that measured ERK phosphorylation levels 1 h after adding vemurafenib. As the figure shows, we used a pERK antibody (marked in green) and a non-specific cell stain (marked in red) to simultaneously monitor ERK phosphorylation and cell confluency in fixed cell cultures. Using this method, we were able to infer the relative ERK activity, normalized to population size, across a range of drug concentrations. Figure 1C shows the drug response curve, as measured by pERK levels, in two melanoma cell-lines harboring the oncogenic BRAF^V600E^ mutation. A375 is a widely studied melanoma cell-line and WM983B is a cell-line that was recently derived from a patient melanoma tumor^5^. We found that the drug has reduced efficacy at a dose of 67.5 nM for A375 cells and 125 nM for WM983B cells (one-tailed t-test with a Bonferroni corrected p-value of 0.005 for ten statistical tests). We concluded that in our experimental system, vemurafenib is a potent signaling inhibitor at sub-micromolar concentrations for both cell-lines.

The previous discovery of signaling reactivation after BRAF^V600E^ targeted inhibition revealed that pERK levels can rebound within a few hours. However, in some cell-lines signaling reactivation can take significantly longer and transpire only after multiple days. In order to validate that signaling reactivation exists in our cells and estimate the timescale required for it to take place, we quantified pERK levels at 24 h intervals over 72 h in both cell lines. Figure 1D shows the results of these bulk population measurements. We observed that for both A375 and WM983B cells maximum inhibition was 1 h after the drug was added and that signaling levels considerably rebounded within 24–48 h reaching an intermediate level (one-tailed t-test with a Bonferroni corrected p-value of 0.01 for five statistical tests). The results for the A375 cell-line agree with previous observations made for this cell-line^13^. Since some recovery already takes place within the first 24 h, we next decided to repeat the experiment and monitor the dynamics of pERK at a high time resolution within the first few hours of adding the drug (Fig. 1E). The experiments revealed that pERK levels decay rapidly after treatment and that inhibition is maximized after an hour. However, the experiments also revealed that signaling inhibition starts to slowly weaken after 2 h of treatment (one-tailed t-test with a Bonferroni corrected p-value of 0.01 for five statistical tests). Taken together our results show that vemurafenib leads to transient signaling inhibition with initial signaling reactivation within 2 h of treatment. This observation is shorter than the previously observed time window of 4 h identified using a Western blot^13^. We note however, that since our ICW approach relies on measurement of pERK level in bulk populations, it is insufficient to discriminate between the signaling rewiring models presented in Fig. 1A.

### Bulk measurement of growth arrest and recovery

**Figure 2:**
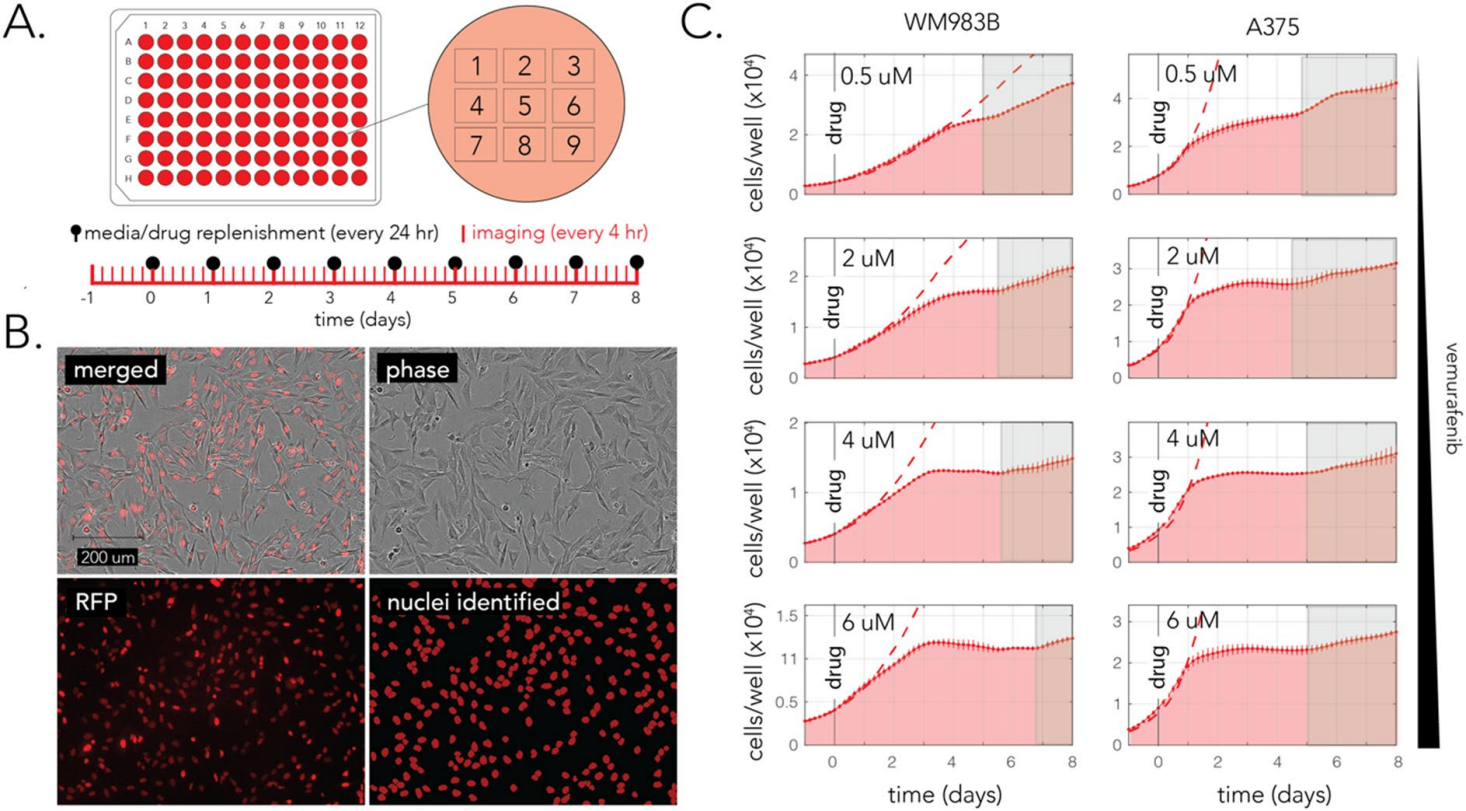
Transient growth arrest after vemurafenib treatment in bulk populations. (**A**) A diagram of the drug treatment and imaging protocol. Cells were plated in a 96-well microplate and drug was replenished every 24 h. Each well was imaged with nine non-overlapping imaging fields and treatments were applied in duplicates. (**B**) Representative microscopy images and the paired image analysis for counting nuclei number in each imaging field. (**C**) Changes in cell number over 8 days of drug treatment across multiple drug concentrations. The red area shows the inferred number of cells (per well) in drug treated wells (the bars show the standard deviation across biological replicates). The dashed red line shows the number of cells in a control (no-drug) experiment. The gray area shows the period with notable growth recovery (determined by eye). We observed growth recovery in both cell-lines, across all drug concentrations.

Cells harboring the BRAF^V600E^ mutation proliferate independently from growth-factors due to their oncogenic mutation. Previous work revealed that although targeted inhibition of the mutated BRAF initially leads to growth arrest, this arrest is transient and that growth-factor dependent proliferation can be later observed^5,13^. We tested if transient arrest existed in the two cell-lines we used by time-lapse microscopy. We reasoned that microscopy observations will allow us to evaluate the magnitude of recovery and evaluate the minimal time interval needed for growth recovery. Towards this aim we used high-throughput time-lapse fluorescence microscopy and quantified changes in the cell numbers by automated image analysis. Quantifying growth by changes in cell number is preferable to using culture confluence since cellular morphology is noticeably influenced by vemurafenib. Figure 2A shows our experimental setup. We plated cells that constitutively expressed a fluorescent nuclear tag at low confluence in a micro-well plate and imaged them over a week-long experiment at 4-h intervals. To minimize potential effects of gradual decreases in drug potency during prolonged incubation due to drug degradation or cell metabolism, we replenished half of the drug-containing media every day. This treatment also ensures that extracellular growth-factors will not be spent by the cultured cells and that dead cells will be regularly removed. Extracellular growth factors were previously shown to be important for growth recovery during vemurafenib treatament^18^. We then used automated image analysis of fluorescence images to calculate the number of individual nuclei in each imaging field. Figure 2B shows representative phase and fluorescence images of a single field of view and a mask image showing the nuclei detected after segmentation of the fluorescence image.

We monitored the growth dynamics of cells in the presence of multiple drug concentrations. We focused on a concentration range that we previously established as fully inhibitory according to pERK levels we observed 1 h after adding the drug (Fig. 1C). Figure 2C shows the results of our experiments across the four drug concentrations in the two tested cell-lines. We observed overall similar trends in growth, arrest, and recovery across all experiment conditions. We observed an almost complete growth arrest, as compared with untreated control cultures within a day or two of drug treatment (marked by a dashed line in Fig. 2C). However, we also detected a slow but reproducible recovery from growth arrest (marked by shaded areas in the graph). The initial response time, as measured by separation between the growth curve of treated and untreated cells, ranged from 24 h in A375 cells to 48 h in WM983B cells. This difference likely originates from the different generation time of the two cell-lines and the time it takes cells to complete the cell-cycle before arresting at the G1 stage due to the drug^21^. Importantly, we observed that in both cell-lines the arrest in growth was transient (1–5 days) and that the arrest period was inversely proportional to drug concentration. Lastly, it was also evident that at least in the experiment’s time period, growth recovery was modest, with resumed growth being substantially slower than the initial growth without the drug. In agreement with previous results showing growth recovery dependance on extracellular growth factors, we also observed a correlation between growth recovery and media replenishment frequency (Supplementary Fig. S1). Taken together, these results confirm that growth arrest is indeed transient, and establish that growth can resume very early after the arrest. Establishing this timeline was key for designing the subsequent time-lapse microscopy experiments that monitored both signaling and growth.

### Single-cell measurement of signaling inhibition and reactivation

**Figure 3:**
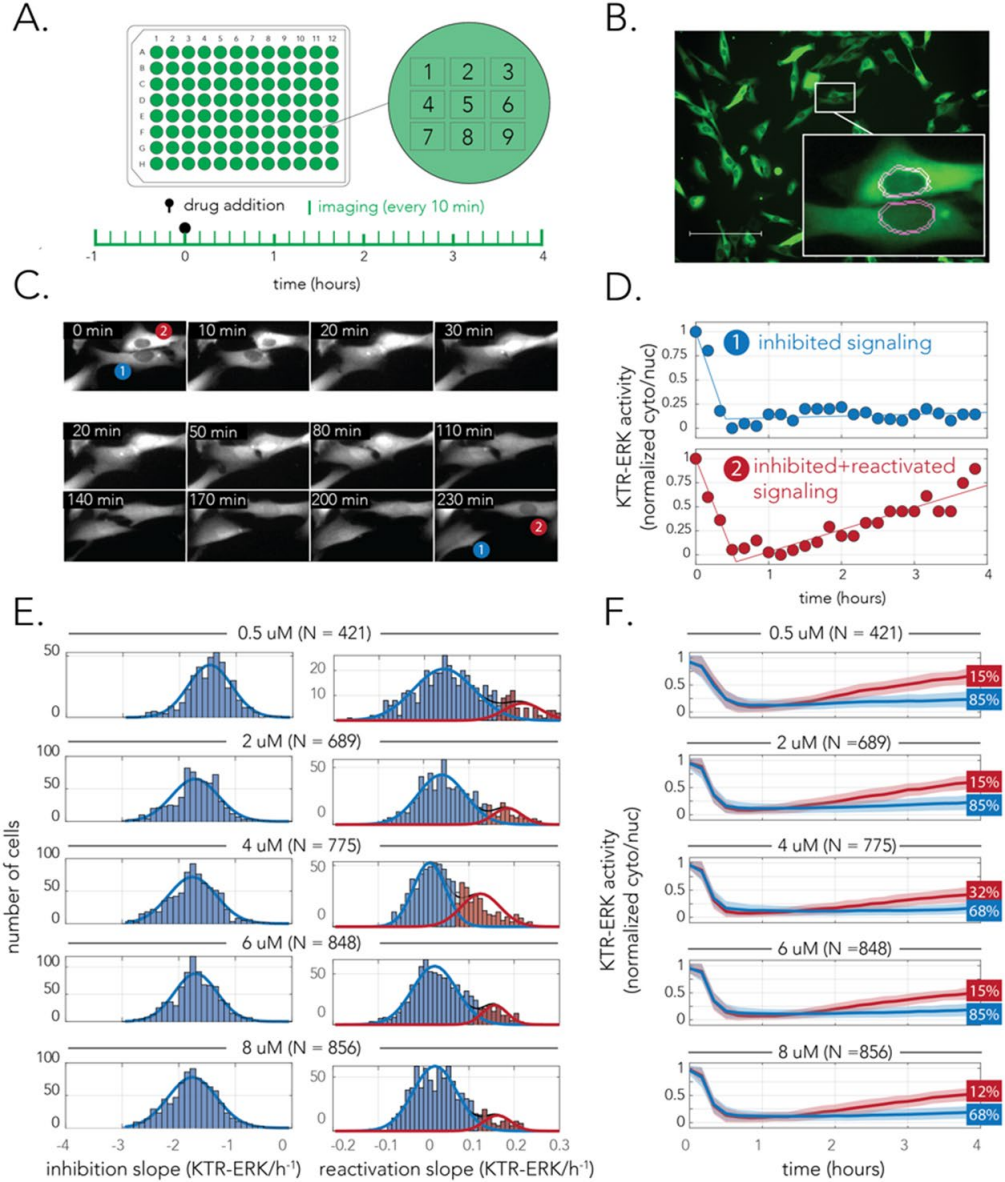
Single-cell measurements of signaling reveal homogenous inhibition yet heterogeneous signaling reactivation in WM983B cells. (**A**) A diagram of the drug treatment and imaging protocol. Cells were plated in a 96-well microplate and were imaged every 10 min over 4 h after drug treatment. (**B**) Representative microscopy image of the translocation fluorescent reporter used to monitor ERK signaling. Image analysis was used to segment both the nucleus region of each cell and a narrow ring surrounding it (to evaluate reporter intensity in the cytoplasm). (**C**) Microscopy images of changes in KTR-ERK in two neighboring cells after drug treatment. Upon 20 min of treatment the fluorescent reporter translocated to the nucleus in both cells (indicating ERK signaling is inhibited). The reporter partially translocated back to the cytoplasm after a few hours in cell #2. (**D**) Quantification of reporter localization in the two cells shown in (**C**) over time reveals different signaling reactivation in neighboring cells. The markers show the ERK activity relative to the range observed during the experiment as determined by the KTR-ERK reporter. The lines show the two-phase linear model that was fitted to the data points collected. Signaling reactivation starts as early as after 1 h in cell #2 while signaling remains inhibited in cell #1. (**E**) Cellular heterogeneity is observed in signaling reactivation but not in initial signaling inhibition. The histograms show the slopes of signaling inhibition (left panels) and signaling reactivation (right panels) across hundreds of co-cultured cells. The histograms were fitted to both unimodal and bimodal gaussians to test for heterogeneity and determine if dynamics were homogenous (unimodal) or heterogeneous (bimodal) with a statistical test. The histograms are colored with one or two colors according to the fit result and the underlying distributions are shown in thin blue and red lines. (**F**) Signaling profiles of all cells grouped and colored according to the bimodal distributions of signaling reactivation. The lines mark the average signaling status for the cell group and the shaded area shows the standard deviation. The numbers at the end of the graphs show the percentage of cells belonging to the cell group.

Bulk measurements of signaling dynamics and cell growth allowed us to outline the relevant period for signaling reactivation and growth recovery (Figs. 1, 2). However, these experiments were insufficient for clarifying the dynamics of individual cells and determining the underlying signaling rewiring model (Fig. 1A). Multiple previous studies by us and many others used translocation-based fluorescent reporters to monitor signaling activity in individual live-cells (e.g.^22,23^). Such reporters alter their subcellular localization in response to a posttranscriptional modification, such as phosphorylation by the kinase of interest, and therefore provide an almost instantaneous marker of the signaling status without hampering cell viability. In order to monitor signaling downstream to BRAF we used a previously developed fluorescent kinase-translocation-reporter (KTR) that localizes primarily in the cytoplasm when ERK is active and pERK level is high^24^ (Supplementary Fig. S2). In contrast, when BRAF activity is inhibited and pERK levels drop, the KTR localizes primarily in the nucleus. Figure 3A shows our experimental setup. We plated cells that constitutively expressed a fluorescent nuclear tag and the KTR-ERK reporter at low confluence in micro-well plates and imaged the cells every 10 min over 4 h after treatment. We decided to conduct these experiments with WM983B cells since the rare subpopulation of resistant cells was well characterized in this cell-line^5^.

We developed an automated image analysis pipeline for monitoring signaling dynamics in individual cells in our time-lapse microscopy experiments. The approach relied on monitoring KTR-ERK fluorescent intensity in each of the imaged cells in addition to tracking cells over consecutive time points of the experiment (see “Methods” section). Figure 3B shows a representative image of cells expressing the KTR-ERK reporter and our approach for image segmentation (Fig. 3B inset). We segmented the nuclear region and a thin ring-shaped region around it to infer the ERK activity from the KTR-ERK intensity in the nucleus and cytoplasm^25^. Figure 3C shows a montage of two cells over the 4 h of the experiment as the KTR-ERK reporter translocated from the cytoplasm to the nucleus in response to BRAF inhibition. As the images show, KTR-ERK translocation can be easily detected within 20 min of adding the drug. Figure 3D shows the signaling dynamics we inferred with our image analysis pipeline for the cells presented in the montage. As the figure shows, although signaling in the two cells had a similar profile in the first hour, they greatly differed in later time points—signaling clearly recovered in cell #2 while it remained fully inhibited in cell #1. In order to capture the temporal dynamics of the signaling profile we fitted the observations made in each cell to a phenomenological model with only three parameters. The signaling dynamics are fitted to a two-stage linear model that capture the slope of signaling inhibition, and the slope of signaling reactivation (or plateaued inhibition). The fitted model for the two cells is represented by the line graphs in Fig. 3D. It is important to note that although more complicated models can surely be used to fit the data, reduction of the dynamics to a two-phase linear model worked very well and was sufficient for our purposes.

The experimental setup we used, coupled with our automated image analysis pipeline, allowed us to simultaneously monitor the signaling dynamics of hundreds of cells in a single experiment. We therefore reasoned that this approach would allow us to identify if signaling dynamics are homogenous or heterogeneous across identically treated cells that are cultured together in a single micro-plate well. Towards this goal we decided to evaluate the regularity of signaling dynamics by inspecting the distribution of the signaling inhibition slopes and the distribution of the signaling reactivation slopes. We expected that homogenous behaviors across the cell population will transpire as unimodal distribution while heterogeneous behaviors will transpire as multimodal distribution. Figure 3E shows the histograms of signaling inhibition and reactivation slopes in five different drug concentrations, all of which are expected to be fully inhibitory by their initial impact (Fig. 1C). We used a Kolmogorov–Smirnov statistical test to see if the histograms better fit a single unimodal gaussian or a bimodal gaussian (mixture of two unimodal gaussians). This analysis revealed that the initial stage of drug response, signaling inhibition, is homogenous in the cell population across the entire drug concentration range. However, the same analysis revealed that signaling reactivation is heterogeneous and fits a mixture of two unimodal gaussian distributions (marked in red and blue lines in the figure). This bimodal distribution indicated that the population consists of two differently behaving subpopulations: signaling remains inhibited for the majority of cells yet it gets reactivated in a minority of them. The signaling dynamics of these two subpopulations is similar to that presented for the two cells in Fig. 3D. Importantly, our analysis also allows us to estimate the proportion of each subpopulation from all imaged cells (marked by the percentage numbers at the edge of the graphs in Fig. 3F). These estimations showed that the proportion of the recovering subpopulation is almost identical across all tested drug concentrations (averaging around 15%).

Taken together, our single-cell results indicate that WM983B cells can be stratified into two subpopulations by their signaling response to vemurafenib within the first few hours of treatment—a large group of cells that remains inhibited and a small group capable of reactivating signaling after initial inhibition within an hour of treatment. These dynamics support the homogenous inhibition and heterogeneous reactivation model of signaling rewiring presented in Fig. 1A. Furthermore, the short time-scale in which the differences between the two subpopulations manifests itself is too short to be explained by a different transcriptional program that is triggered post-treatment. The differences in signaling rewiring therefore likely reflect dissimilar transcriptional states that pre-existed the drug. This hypothesis is further supported by the observation that the same fraction of cells recovered irrespectively from the drug concentration used.

### Single-cell measurement of growth recovery

Our long-term measurement of bulk population growth after drug treatment revealed that the cell cultures are transiently arrested and then resume growth at a slow rate (Fig. 2C). Given the cell–cell heterogeneity we observed in signaling reactivation, a key question that arises is whether the signaling profile of a cell within hours of drug treatment is a good predictor of its proliferation capacity. To address this question, we combined the experimental approaches we used previously (Figs. 2, 3) in a two-phase time-lapse microscopy experiment. In this experiment we first monitored early signaling dynamics, at a high time resolution, and then continued and monitored the proliferation of individual cells over 7 days at a lower time resolution (see “Methods” section). Since accurately tracking individual cell lineages over multiple days was impractical by automated image analysis (due to cell movement, division, and detachment) we tracked lineages by eye after observing time-lapse microscopy images.

**Figure 4:**
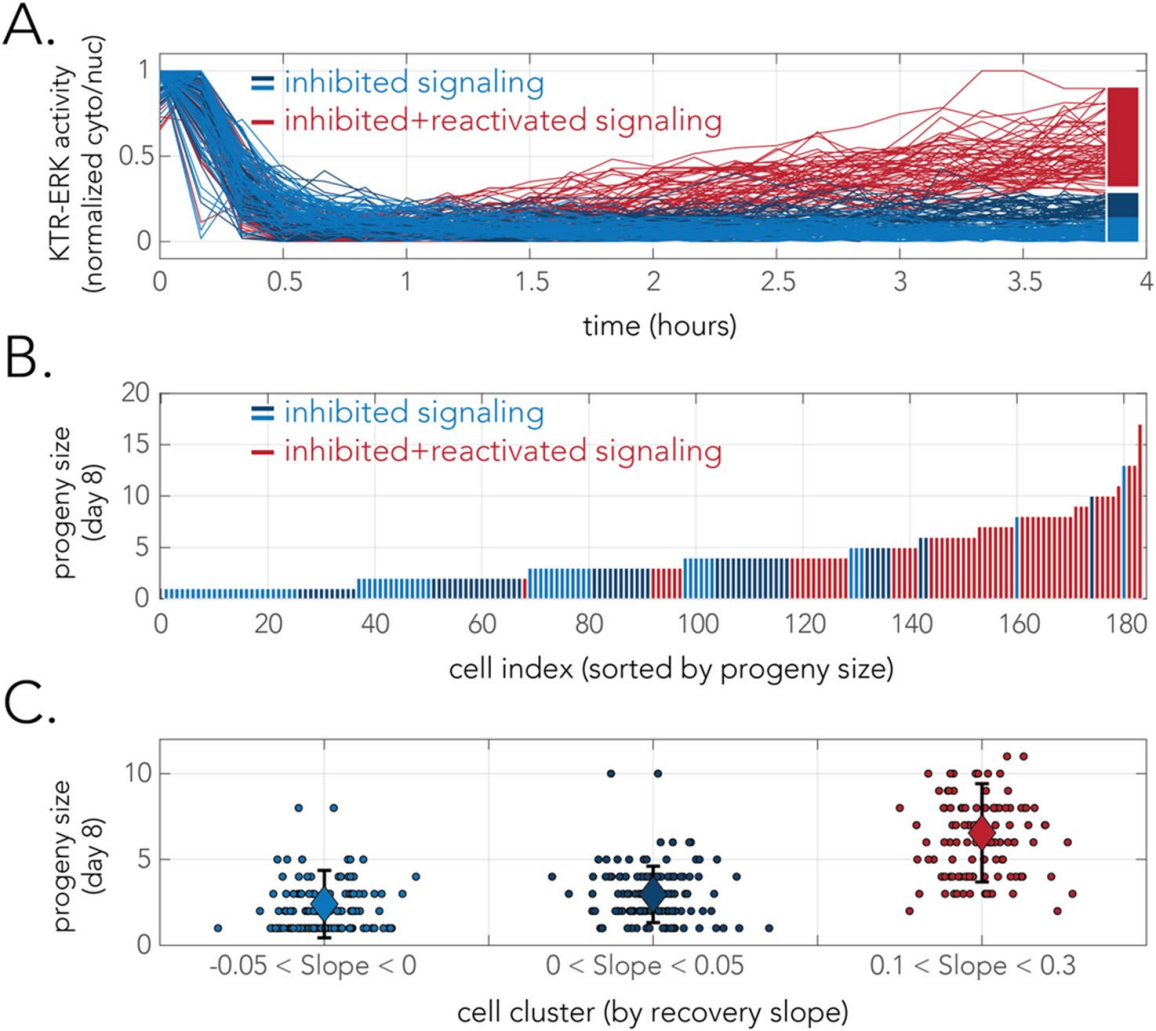
Single-cell measurements connect early signaling recovery with long term drug resistance. (**A**) Signaling dynamics at the first 4 h after drug treatment of 180 cells treated with inhibitory concentration of vemurafenib (6 uM). The colors mark the signaling recovery capacity of the cells (recovering cells in red and non-recovering cells in blue). The identity of the cells was determined solely according to the slope of the signaling curve after the initial inhibition phase. The non-recovering cells were further subdivided to strongly inhibited cells (light blue) and weakly inhibited cells (light blue). (**B**) Lineage sizes descending from the 180 cells after seven days of drug treatment. The bars show the lineage descending from single cells and the bar colors mark initial signaling recovery of the ancestor cell. (**C**) Lineage sizes descending from ancestor cells according to the signaling recovery group. Each point marks the lineage descending from a single ancestor cell and the large diamond markers show the average lineage size. Error bars represent standard deviation.

In these experiments we focused on the WM983B cells treated with a single inhibitory concentration of vemurafenib throughout the experiment (6 uM) and focused on a representative sample of 180 cells chosen by their early signaling dynamics. Figure 4A shows the signaling dynamics of the subset of cells that we chose for this analysis (taken from two independent experiments). A sample group of 120 cells was chosen to represent the major subpopulation of non-recovering cells (blue lines in Fig. 4A). This group was further subdivided into a group of 60 fully inhibited cells (light blue lines) and a group of 60 inhibited cells with a marginal recovery (dark blue lines). A sample group of 60 cells was chosen to represent the minor subpopulation of signaling reactivating cells (red lines in Fig. 4A). We next counted how many cells descended from the 180 monitored cells during a period of seven days of drug treatment (a period sufficient to detect growth recovery, Fig. 2C). Figure 4B shows bar plots representing the lineage size originating from each tracked cell after sorting the cells by their number of decedents and coloring the bars according to the early signaling dynamics. As the figure shows, we observed that cells characterized by early signaling recovery (red bars) had significantly larger lineages than non-recovering cells (blue bars). A non-parametric statistical test on the rank order of cells from the two groups rejected the null hypothesis that they are characterized by identical lineage sizes (p-value < 10^–18^, Wilcoxon Rank Sum Test). The significant correlation between early signaling dynamics and lineage size can also be visualized by comparing the average number of decedents of cells from each group (Fig. 4C), with recovering cells giving rise to an average lineage size of 6.6 cell within a week of drug treatment and non-recovering cells giving rise to an average 2.7 cells in the same period. A statistical test considering the sample groups rejected the hypothesis that the two groups have similar lineage sizes (p-value < 10^–20^, two-tailed t-test). It is important to note the fact that even non-recovering cells are not completely arrested (lineage size larger than one) is compatible with the observation that growth arrest is not immediate with drug treatment (Fig. 2C) and that growth arrest due to vemurafenib takes place at the G1 stage^21^. Taken together the results of this experiment establish a direct association between the early reactivation of signaling and ultimately cell proliferation capacity. These direct observations of individual cells throughout the treatment period strongly supports the heterogeneous model of drug recovery presented in Fig. 1A.

## Discussion

Phenotypic differences between cells are a defining characteristic in cancer that greatly increases disease complexity and challenges its treatment. Numerous studies over the past decades established that cell-to-cell heterogeneity can engage key oncogenic pathways underlying disease and can therefore pose significant challenges for personalized cancer medicine^1^. While much attention is currently dedicated to uncovering phenotypic diversity that arises due to genetic differences between cells in the same tumor, recent works uncovered that epigenetic mechanisms, operating differently within isogenic cells, also play a significant role in promoting heterogeneity with clinical implications^2^. Here we focused on a widely used in-vitro model for studying adaptive drug resistance—growth recovery after treatment with a targeted therapy (vemurafenib) against a common oncogenic BRAF mutation^13^. We chose this system since previous work indicated that it features highly heterogeneous drug resistance and pointed to a plausible underlying epigenetic mechanism—differences between isogenic cells in the expression of key genes in the cellular network responding to growth factors^5^. Although such transcriptional differences are potentially compatible with the widely accepted model of vemurafenib resistance through post-treatment reliance on extracellular growth signals^13,18^, direct evidence of this connection is currently missing. Specifically, there are no observations of heterogeneity in signaling reactivation at the single-cell level and no evidence of an association between the profile of signaling of individual cells and their proliferation capacity during drug treatment^23,26–29^.

It is important to note that there is a considerable challenge in gathering such direct evidence of a connection between cellular processes that manifest on such different time scales that might also be highly heterogeneous across the population. First, work on heterogeneity requires single-cell assays that are typically more demanding than bulk population measurements and typically rely on “end-point” assays that require fixing the cells (e.g., immunofluorescence) or at least significantly perturbing them (e.g., live-cell cytometry). Due to this limitation, multiple independent observations, as signaling and subsequent proliferation, can rarely be done on the same exact cell. Additionally, in this specific model system, there is the need to simultaneously monitor multiple cellular processes, from cell-to-cell differences in the transcriptional program that exist for days before the drug treatment^5^, followed by rewiring of the signaling network that can transpire within minutes of drug treatment, and eventually with changes in cell proliferation that take multiple days^13^. Indeed, this challenge of bridging events that transpire on multiple time-scales is common to many model systems in cell biology^30^. Here we addressed this challenge by performing time-lapse microscopy experiments, at different time resolutions, in a week-long experiment while maintaining ideal growth conditions (without any perturbations to the treated cells). This methodology has proven valuable in bridging such time gaps between a fast signaling event, transcriptional response and cell fate decisions in the past (e.g.^23,26–29^). While being demanding, this approach allowed us to make single-cell observations and gather direct evidence establishing a clear connection between early signaling dynamics and long-term proliferation capacity. These observations fill in a critical piece of the puzzle that connected the widely accepted rewiring model, suggested a decade ago, to population heterogeneity in drug sensitivity that was reported only very recently^5^.

The experimental approach we present here can be further used to address additional open questions in this important model system. For example, while we established a connection between signaling dynamics and drug resistance, additional work will be required to establish a connection between the transcriptional state of cells pre-treatment and their signaling dynamics post-treatment. However, this connection may not be easy to establish given that cells existing in the resistant state prior to the drug may themselves be highly heterogeneous, with different resistant cells over-expressing a different set of resistance genes^5^. Another important question that might be addressed using our microscopy approach concerns the stability and inheritance of the resistance state. For example, it is possible to monitor if kin-cells that descended from a common ancestor prior to treatment share a common signaling profile as a function of the time that elapsed from cell division to drug treatment. Such measurements can help refine parameters of the state model previously developed for these melanoma cells^5^. Alternatively, information regarding other signaling pathways can also be collected using orthogonal live-cell reporters in order to provide direct evidence of additional early signaling events that predict long-term drug resistance. Such investigation is highly interesting in the context of the JNK pathway that was previously suggested to be implicated in vemurafenib drug resistance^12,14^.

## Methods

### Cell-lines and media and antibodies

A375 (ATCC) and WM983B (Arjun Raj lab) cells were cultured in DMEM supplemented with 5% fetal bovine serum (Gibco FBS cat# 26140-079) and incubated at 37 °C with 5% CO_2_. All experiments were conducted by plating cells on 96-well plates 12–18 h before starting the experiment. BRAF targeted inhibition was achieved with Vemurafenib (Selleckchem, PLX4032). Time lapse microscopy experiments were performed with cells constitutively expressing fluorescent reporters after infection with lentivirus.

### Live-cell fluorescent reporters

Plasmid constructs for mammalian expression were cloned into the pHR lentiviral backbone similarly to our previous work^22^. We cloned the mammalian histone H2B-mRuby and the pathway reporter KTR(ERK)-mEGFP^24^ into separate plasmids. Lentivirus was produced by co-transfecting the pHR plasmids and vectors encoding packaging proteins (pMD2.G and p8.91) using the Fugene 6 HD transfection reagent in HEK-293T cells plated in 6-well plates at ∼ 70% confluency. Viral supernatants were collected 2 days after transfection and 0.45 μm filtered and used for transduction immediately. A375 and WM983B cells were cultured in 5% fetal bovine serum in DMEM at 37 °C with 5% CO_2_ in a humidified incubator. For viral transduction, cells were plated in 6-well plates to achieve ∼ 20% confluency at the time of infection. For lentiviral transduction, we added 50 μL of virus supernatant directly to cells. Viral media was replaced with growth media 24 h post infection.

### 72-h dose response (ICW)

A375 and WM983B were seeded in 96-well plates (Greiner, 655090; 7500 cells/well) in 100 uL media the day before treatment. Vemurafenib (Selleckchem, PLX4032; 2× in 100 uL media) was added to wells in triplicate with doses 0 uM, 0.031 uM, 0.062 uM, 0.125 uM, 0.25 uM, 0.5 uM, 1uM, 2 uM, 4 uM, 8 uM for 1 h. Vemurafenib media was removed and wells were washed 3 times with fresh media before replenishing wells with 200 uL media. After 72 h, cells were analyzed with an in-cell western.

### pERK peak inhibition and recovery (ICW)

A375 and WM983B were seeded in 96-well plates (Greiner, 655090; 10,000 cells/well) in 100 uL media in triplicate the day before treatment. Vemurafenib (Selleckchem, PLX4032; 2× in 100 uL media) was added for a final concentration of 0.5uM. Cells were treated for 0, 0.25, 0.5, 1, 2, and 4 h (peak inhibition and short-term recovery) and for 0, 1, 24, 48, and 72 h (long-term recovery) before analyzing with an in-cell western.

### In-cell western

Cells were grown and treated in 96-well plates. Treated cells were fixed with 3.7% formaldehyde for 20 min at room temperature (RT) and washed 2 times with 200 uL PBS (RT). Cells were permeabilized with 200 uL/well 0.5% Triton X-100 (10 min, RT) and blocked with 150 uL/well Odyssey Blocking Buffer (LI-COR) for 1.5 h at RT with 300 rpm shaking. Cells were incubated overnight at 4 °C with anti-phospho-Erk1/2 (Cell Signaling #4370; 1:400). Primary antibody solution was removed and wells were washed 5 times with 200 uL PBS-T (5 min each, RT, 300 rpm). A near-infrared (800 nm) fluorescent secondary antibody (LI-COR 926-32210; 1:800) and near-infrared (700 nm) cell counterstain (LI-COR 926-41090; 1:2000) were incubated with cells (1 h, RT, 300 rpm). Secondary antibody was removed before washing wells 2 times with 200 uL PBS-T (5 min each, RT, 300 rpm). 96-well plates were imaged with LI-COR Odyssey. Signal (800 nm and 700 nm) per well was quantified using Image Studio Lite (LI-COR). Relative phospho-Erk1/2 signal per well was determined by dividing the phospho-Erk1/2 signal (800 nm) by the CellTag signal (700 nm) and replicates averaged (mean).

### Time-lapse microscopy and automated image analysis

For imaging, cells were plated in 96-well TC-treated plates (Eppendorf) at a concentration of 3000 cells/well and were allowed to adhere over night before imaging started (12–18 h). All microscopy images were obtained using an IncuCyte S3 microscope platform (Sartorius) that is placed inside a humidified incubator with temperature and CO_2_ control. We used built-in filter sets in the IncuCyte microscope (Green/Red 4616 optical module) to monitor GFP (300 ms acquisition time), RFP (400 ms acquisition time) and phase channels. For experiments monitoring cellular signaling we imaged nine none-overlapping fields in each well with a × 20 magnification objective with time intervals of 10 min. For experiments monitoring cell growth, we imaged nine none-overlapping fields in each well with a × 20 magnification objective with time intervals of 1 h. All experiments were conducted with biological replicates in separate wells (indicated in the “Results” section).

Image analysis was performed similarly to our previous work^22^ with the propriety software of the IncuCyte microscope and additional custom written computer scripts developed in MATLAB (Mathworks). Calculation of nuclei number in each imaging field was based on segmentation of the mRuby fluorescence signal with the IncuCyte software. We used Top-Hat algorithm to correct for uneven illumination. We used area and eccentricity filters to further remove fluorescent objects that are not live cells (specs of dust, cellular debris). The number of nuclei per well was calculated by summing the number of objects detected over all imaging fields and multiplying a constant conversion coefficient (the coefficient is the ratio of well surface area over the total area of the imaging fields).

To analyze signaling dynamics we first segmented the nuclei according to the mRuby fluorescence signal using the IncuCyte software and produced black/white mask images that correspond to the detected nuclei. These mask images were first used for registration of fluorescence images (aligning consecutive images to account for systematic drift) and then used for segmenting individual cells and inferring the signaling activities (inferring the localization of the nucleus region and cytoplasm ring region and in the signal intesity^22,25^). For image registration we used the Descriptor based registration plugin^31^ in the Fiji distribution^32^ of ImageJ^33^. Registered images were then used as input to a Matlab script that segmented all the objects (nuclei) in each mask image and a narrow ring region around the segmented nuclei. We subtracted the background fluorescence from GFP images and measured the 80th percentile nuclear intensity and the corresponding cytoplasm ring region. Pathway activity was calculated as the ratio between the signals of the cytoplasm ring and the nuclear region^22^. Lastly, in order to track temporal changes in signaling activity, we reconstructed continuous pathway activity for each cell over the entire time-lapse period (4 h) by tracking individual cells over time. Cell tracking was performed with a Matlab script that assigned to each nucleus the spatially closest nucleus over consecutive time points while filtering out nuclei that move to far or underwent significant changes in their area (typically corresponding to cells undergoing mitosis). By assigning highly conservative filters for removing suspicious nuclei we were able to maintain highly reliable tracks for over half of all detected nuclei (estimated by inspection of tracks for individual random cells). Nuclei that were filtered out were ones that migrated out of the imaging area, underwent mitosis or lost adherence during the experiment.

## Supplementary Information

Supplementary Information.
