# Supplementary material for: Rapid signaling reactivation after targeted BRAF inhibition predicts the proliferation of individual melanoma cells from an isogenic population
Source: Sci Rep. 2021 Jul 29;11:15473. doi: 10.1038/s41598-021-94941-8 (PMC8322260; doi:10.1038/s41598-021-94941-8)
Supplement: Supplementary file 1 — Supplementary Information. [file 41598_2021_94941_MOESM1_ESM.docx]

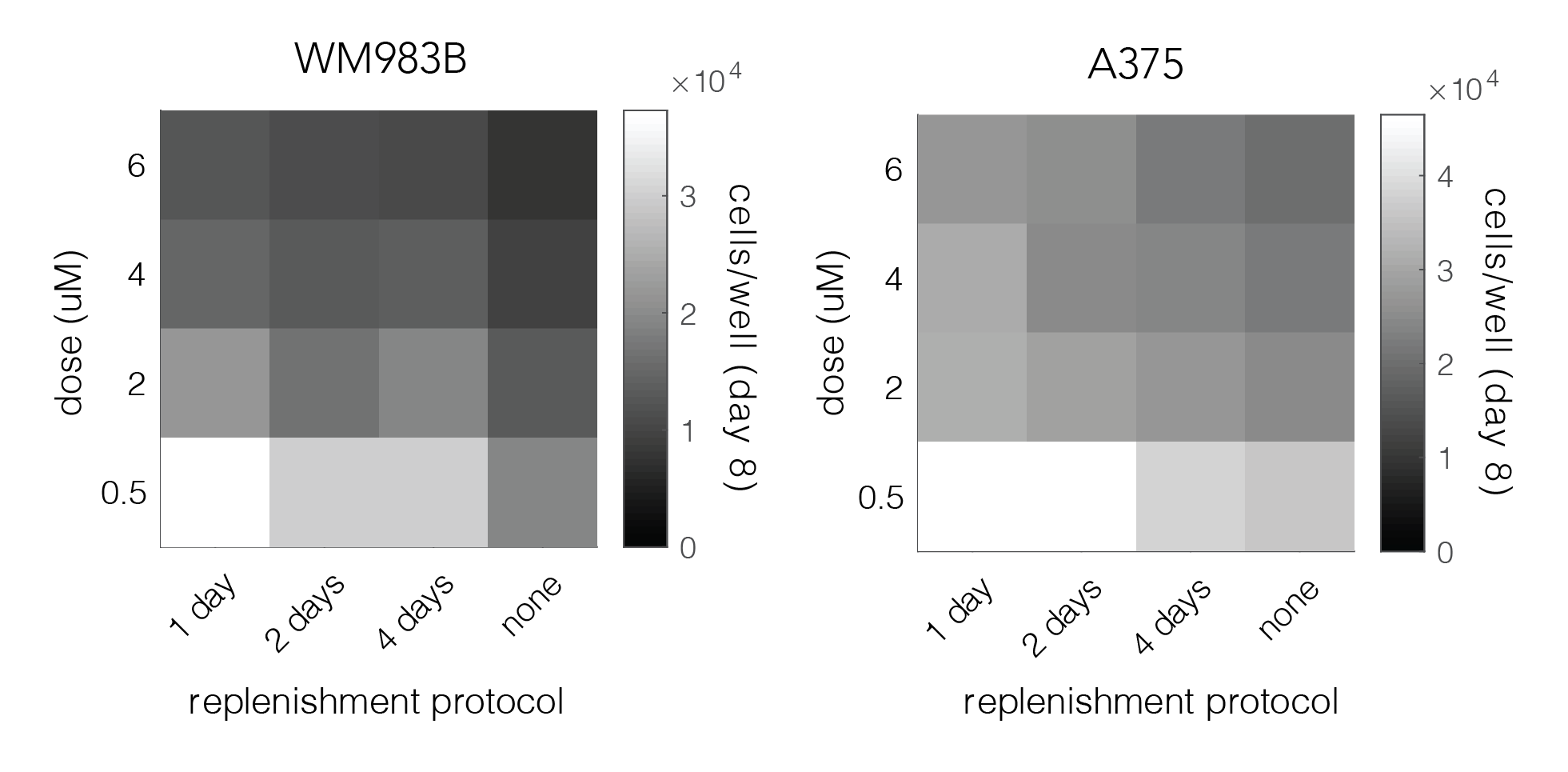


**Supplementary Figure 1. Growth recovery depends on drug concentrations and media replenishment protocol.** The heatmaps show the number of cells observed after eight days of drug treatment. In both cell-lines we observed that culture growth (drug resistance) was inversely correlated with the drug concentration and positively correlated with frequency of replenishment (half the well volume was replenished with new drug-containing media). The grayscale reflects the average cell numbers counted in two biological replicates (as described in the main text).


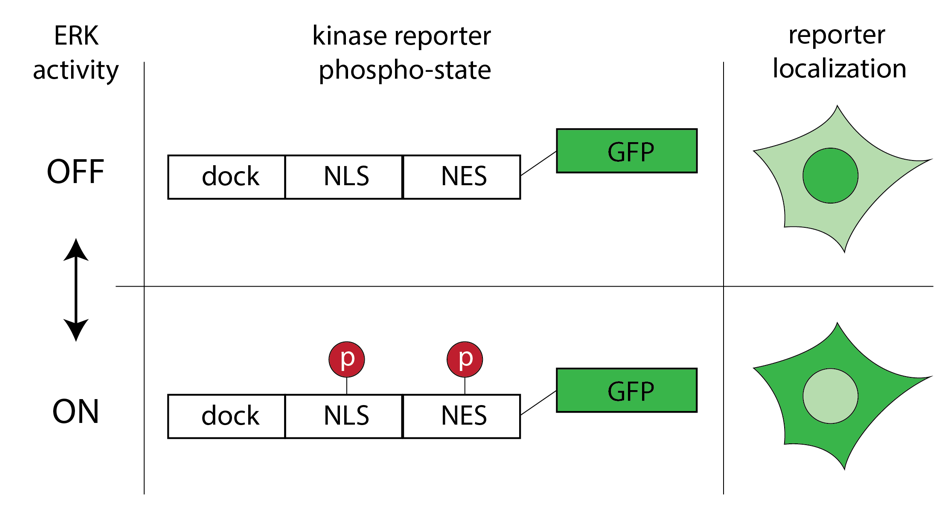


**Supplementary Figure 2. The molecular mechanism underlying the kinase translocation reporter (KTR) previously developed by the Covert lab and used in this study.** The reporter was constructed by fusing a kinase docking domain with a nuclear localization sequence (NLS), a nuclear exit sequence (NES), and a fluorescent protein (GFP). Active ERK (pERK) binds to the docking domain of the reporter and phosphorylates the NLS and NES. Phosphorylated NLS and NES favor the translocation of the reporter to the cytoplasm.


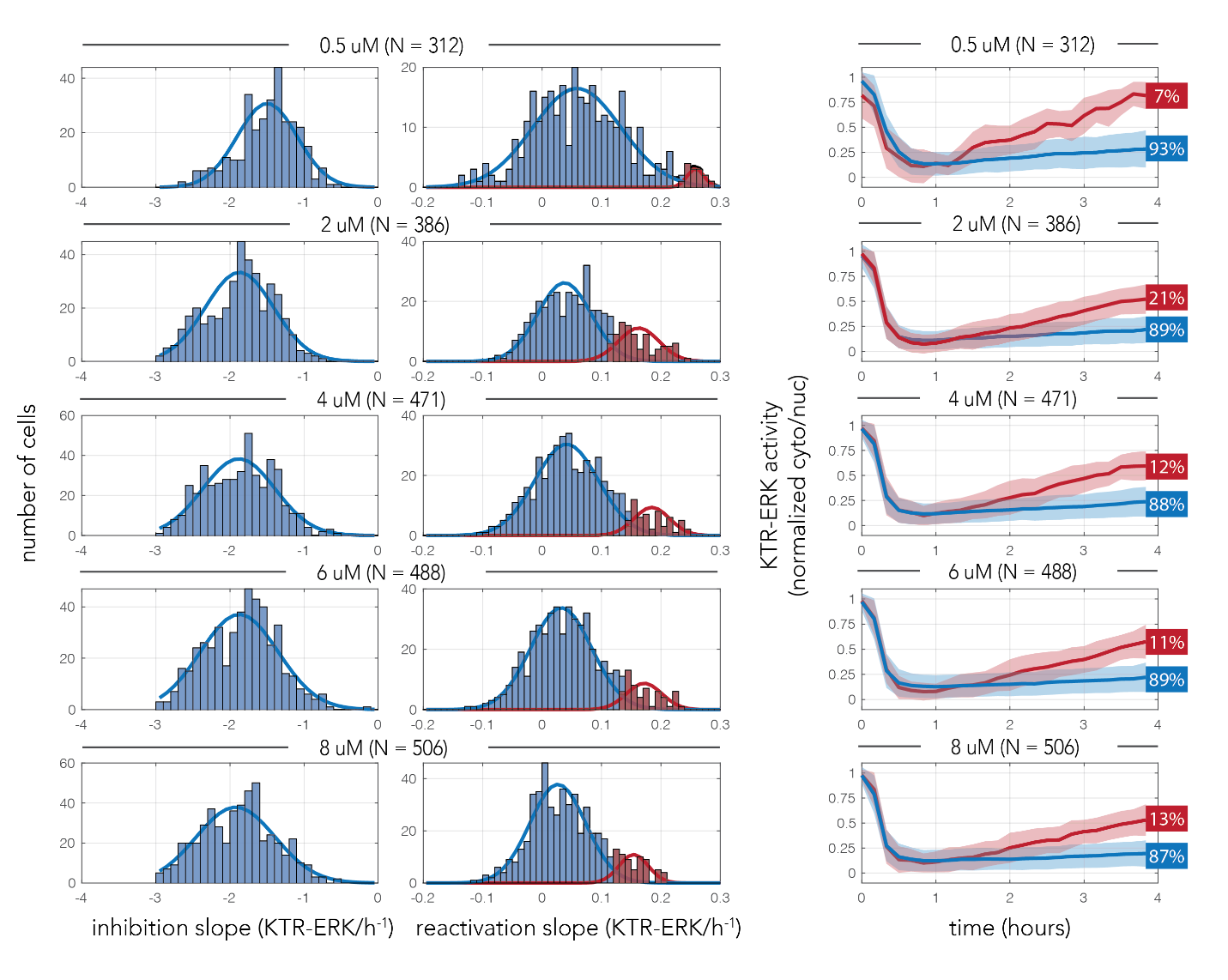


**Supplementary Figure 3. Biological replication of the experiment presented in Figure 3.** (A) Cellular heterogeneity is observed in signaling reactivation but not in initial signaling inhibition. The histograms show the slopes of signaling inhibition (left panels) and signaling reactivation (right panels) across hundreds of co-cultured cells. The histograms were fitted to both unimodal and bimodal gaussians to test for heterogeneity and determine if dynamics were homogenous (unimodal) or heterogeneous (bimodal) with a statistical test. The histograms are colored with one or two colors according to the fit result and the underlying distributions are shown in thin blue and red lines. (B) Signaling profiles of all cells grouped and colored according to the bimodal distributions of signaling reactivation. The lines mark the average signaling status for the cell group and the shaded area shows the standard deviation. The numbers at the end of the graphs show the percentage of cells belonging to the cell group.


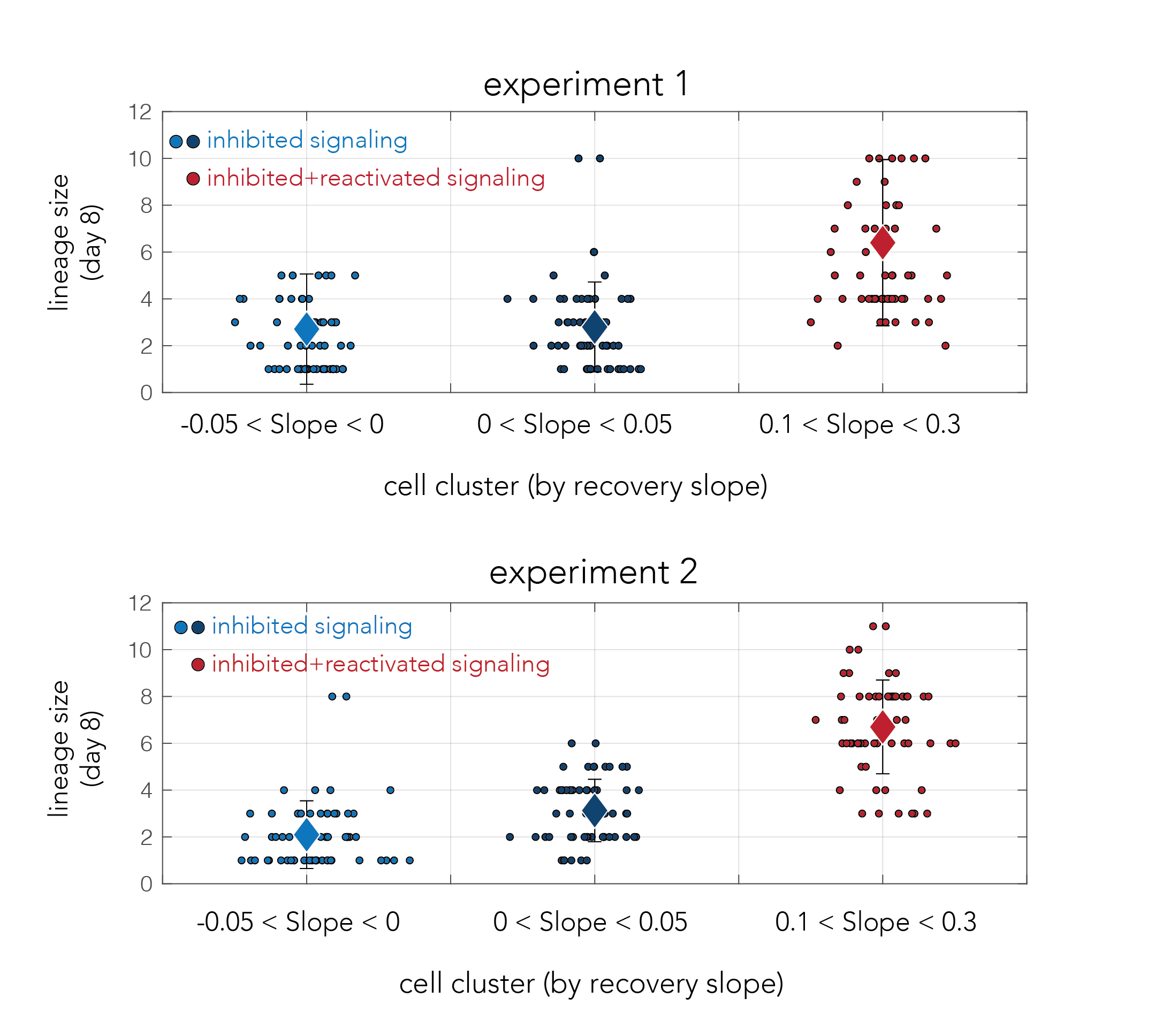


**Supplementary Figure 4. Biological replicates used in the experiment presented in Figure 4.** The top and lower panels show the observations made in two separate experiments (performed on different days) that measured the correlation between early dynamics of ERK activity and the long-term proliferation of individual cells. The y-axis shows the lineage sizes descending from ancestor cells according to the signaling recovery group. Each point marks the lineage descending from a single ancestor cell and the large diamond markers show the average lineage size. Error bars represent standard deviation.
